# Entropy Governs the Structure and Reactivity of Water Dissociation Under Electric Fields

**DOI:** 10.1021/jacs.5c12397

**Published:** 2025-09-25

**Authors:** Yair Litman, Angelos Michaelides

**Affiliations:** ‡ Max Planck Institute for Polymer Research, Ackermannweg 10, Mainz 55128, Germany; † Yusuf Hamied Department of Chemistry, 2152 University of Cambridge, Lensfield Road, Cambridge CB2 1EW, U.K.

## Abstract

The response of water to electric fields is critical to the performance and stability of electrochemical devices, and the selectivity of enzymatic, atmospheric, and organic reactions. A key process in this context is the water (auto)­dissociation reaction (WD), which governs acid–base aqueous chemistry and shapes reaction rates and mechanisms. Despite its significance, the thermodynamics of the WD reaction in electrified environments remains poorly understood. Here, we investigate the WD reaction under external electric fields using ab initio molecular dynamics simulations within the framework of the modern theory of polarization. Our results reveal that strong electric fields dramatically enhance the WD reaction, increasing the equilibrium constant by several orders of magnitude. Moreover, we show that the applied field transforms the WD reaction from an entropically hindered process to an entropy-driven one. Analysis shows that this is because the electric field alters the tendency of ions to be structure makers or structure breakers. By highlighting how strong electric fields reshape solvent organization and reactivity, this work opens new avenues for designing aqueous electro-catalysts that leverage solvent entropy to enhance their performance.

## Graphical abstract

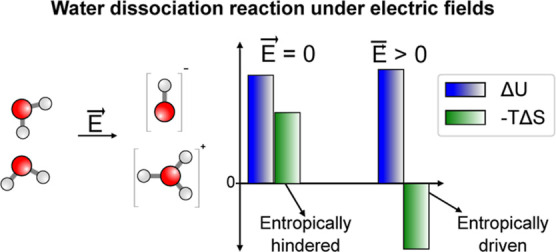

## Introduction

The catalytic effect of electric fields in chemical reactions is a well-established and ubiquitous phenomenon. Many enzymes rely on localized fields to lower activation barriers,^1–3^ electrochemical redox processes fundamentally depend on electric-field-driven charge transfer,^4^ and even nonredox organic reactions can be substantially accelerated in the presence of oriented-external electric fields.^5–9^ In aqueous environments, large electric fields can arise naturally due to molecular fluctuations, hydrogen-bond dynamics, and charge transfer processes.^10–12^ These fluctuations are believed to be enhanced in anisotropic environments, such as interfaces, with estimated intensities reaching up to 0.1 V/Å, even though the associated average field magnitudes remain significantly smaller.^13^ Such field enhancement has been invoked to rationalize the phenomenon of “on-water catalysis”.^14–16^ However, several studies have demonstrated that the observed reactivity enhancements are not always attributable to electric fields,^17–19^ and the role of interfacial electric fields in water catalysis remains an actively debated topic.

An elemental reaction sensitive to electric fields is the water (auto)­dissociation (WD) reaction, 2H_2_O → H_3_O^+^ + OH^–^. This reaction is fundamental to aqueous chemistry, forming the basis of all acid–base equilibria. Several theoretical studies have investigated water dissociation (WD) in bulk water.^11,20–22^ It is generally accepted that transient electric fields play a crucial role in destabilizing the covalent O–H bond, initiating autoionization. The resulting ion pair is then separated by the Grotthuss mechanism to distances of approximately 7–8 Å. Subsequent solvent fluctuations that disrupt the hydrogen-bond network connecting the ions further hinder rapid recombination. More recently, WD has been explored in anisotropic environments, such as the water/air interface^23^ and under nanoconfinement.^24–26^ These studies have highlighted the impact of various factors such as the relative stabilization of the proton and hydroxide, pressurization effects, and interfacial chemistry on the dissociation process. However, the behavior of WD and its underlying thermodynamics under strong electric fields remains much less understood.^27–32^ In a recent study, Cai et al.^33^ studied the graphene–water interface and elegantly correlated the interfacial electric fields with WD reaction rates. They observed pronounced rate enhancements at fields exceeding 0.01 V/Å, consistent with earlier ab initio simulations in bulk water that identified a dissociation threshold near 0.3 V/Å.^30^ The acceleration of water dissociation rates with increasing electric field strength is intuitive: electric fields stabilize the separation of charged fragments that lead to larger dipole moments. Both experiments and simulations consistently confirm this trend. However, a systematic investigation and fundamental physical understanding of the thermodynamics of this process under electric fields has remained elusive.

Understanding the WD reaction at finite electric fields is a fundamental prerequisite to elucidating more complex reactions, since small field-induced shifts in the autoionization equilibrium can lead to large changes in local H^+^ or OH^–^ concentrations, substantially altering reaction rates and mechanisms. By studying the WD reaction in bulk, where interfacial effects are absent by design, one could establish an important baseline against which more complex interfacial phenomena can be rigorously assessed. In this work, we conduct extensive ab initio molecular dynamics (AIMD) simulations to probe the thermodynamics of the WD reaction across a range of temperatures and applied field strengths. By quantifying the reaction energy (Δ*U*) and entropy (Δ*S*) as functions of the external field, we find that Δ*U* remains large and positive with only a minimal dependence on field strength, contrary to common expectations based on simple electrosctatic arguments. Strikingly, the reaction shifts from being entropically hindered at zero field to entropically driven at a field of 0.36 V/Å. We attribute this behavior to structural changes in the hydrogen-bond network, arising from the interplay between field-induced ordering and ion-mediated disruption. Entropic contributions are often overlooked in computational electrocatalysis studies that rely on static calculations to estimate reaction energetics under bias.^34,35^ Our findings show that neglecting entropic changes can lead to qualitatively incorrect conclusions when studying aqueous reactions. At the same time, entropy could serve as a previously underexplored dimension for understanding aqueous reactivity in strong electric fields and for guiding the design and optimization of aqueous electrochemical systems.

## Results and Discussion

### External Electric Fields Reduce the Water pKw

Finite electric fields in periodic density functional theory (DFT) can be treated within the Modern Theory of Polarization.^36–39^ Within this framework, the system’s response to an electric field, **E**, is described by the electric enthalpy functional^40–42^ defined as

1 $$F\left(\nu ,E\right)=E_{KS}\left(\nu \right)-\Omega E\cdot P$$

where ν represents the ionic and electronic degrees of freedom, *E*_KS_ is the Kohn–Sham energy, Ω the cell volume, and **P** the Berry-phase polarization.^36,37,43^ The electric field appearing in eq 1 is the macroscopic (Maxwell) electric field, which is distinct from the applied electric field **E**_0_. The difference between the two is the polarization field generated by the polarization of the water molecules.^42,44^ We performed AIMD based on DFT for bulk liquid water in the NVT ensemble at 330 K using the revPBE exchange–correlation functional^45^ augmented with D3 dispersion corrections^46^ (Figure 1a). Simulations were carried out across a range of different field strengths, up to **E** = 0.4 V/Å. We note that the electric field, **E**, as appears in eq 1, plays the role of a pair of virtual electrodes at infinity.^47^ Thus, we model an idealized situation in which the simulated system is infinitely large and interfacial effects are negligible. Further methodological details, including sensitivity tests with respect to the exchange–correlation functional and system size, are provided in the Methods section and Supporting Information.

**Figure 1:**
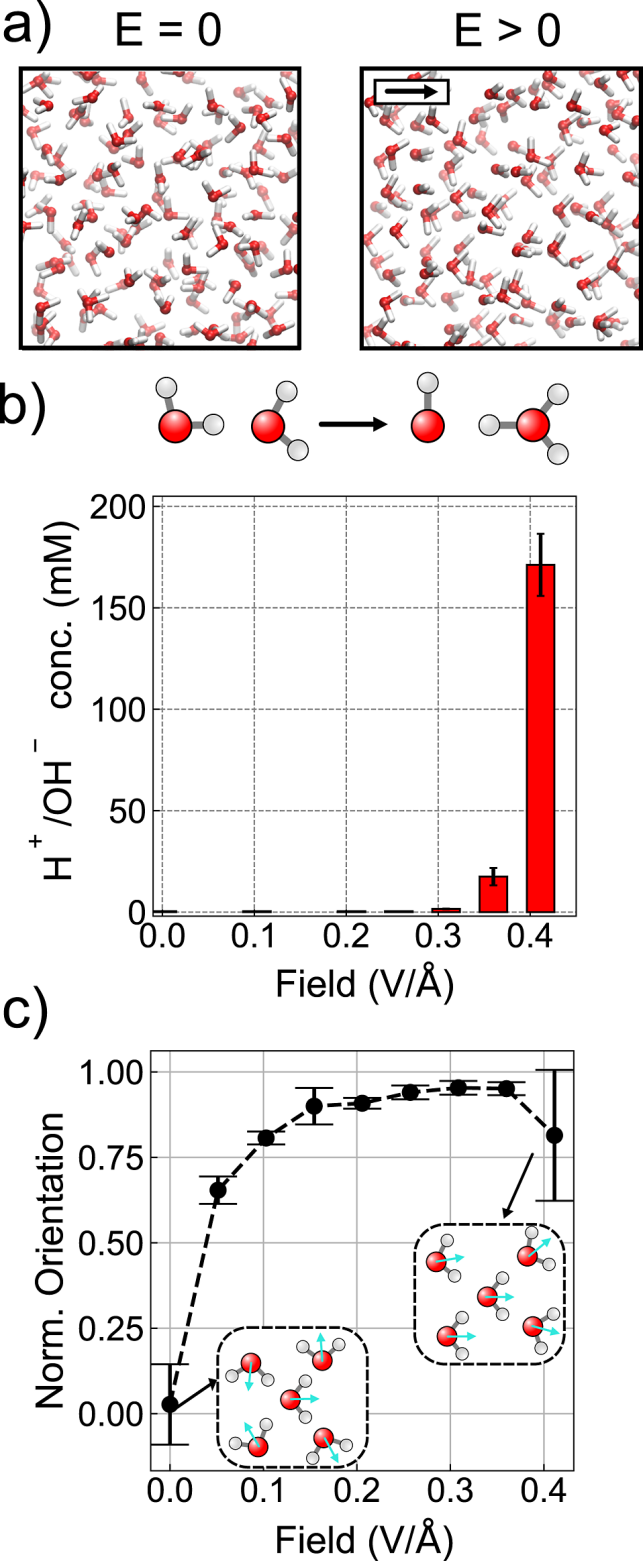
Reactivity and structuring of water under electric fields. (a) Schematic representation of the model system in the absence (left) and presence (right) of an external electric field. The external electric field is coupled to the system through the electric enthalpy functional (see main text) and leads to a net polarization. The arrow in the right panel inset depicts the field direction. (b) Proton defect concentration as a function of the field strength. The threshold for the detection of WD reaction events in the employed setup is **E** = 0.3 V/Å. (c) Average orientation of molecular dipoles along the field direction for pure water. A value of 1.00 represents a perfect alignment between the molecular dipoles and the external field.

Figure 1b shows the equilibrium proton and hydroxide concentration for the different simulations. In agreement with previous studies,^30,31^ we observe WD events above a threshold of **E** = 0.36 V/Å, above which, the number of ions dramatically increases, reaching a value of 170 ± 15 mM at **E** = 0.41 V/Å. These concentrations represent time-averaged values, as the proton and hydroxide species are short-lived, with subpicosecond lifetimes before undergoing rapid recombination.^48^

The autoionization constant, pKw, can be readily computed at a field strength of **E** = 0.36 V/Å or higher from the average ion concentrations of proton and hydroxide ions. However, below this threshold, dissociation events are not observed during the time scales of our simulations, necessitating the use of enhanced sampling techniques to drive the reaction. Several collective variables and advanced sampling techniques have been developed to address this challenge.^20–23,49–51^ In this work, we employed the umbrella integration scheme^52,53^ with a single collective variable constructed from the coordination number, *n*_cov_, of a selected water molecule that smoothly transitions from reactant (*n*_cov_ ∼2) to products (*n*_cov_ ∼1) representing intact water and hydroxide species, respectively.^22,49^ The first two columns of Table 1 provide the values of pKw at selected field strengths. Under zero-field conditions, our simulations predict a value of 14.7 ± 0.3, in good agreement with experiments.^54^ As the field increases, the pKw decreases monotonically, reaching a value of 2.2 ± 0.2 at **E** = 0.41 V/Å, the highest considered field strength.

**Table 1:** pKw and Free Energy Decomposition of WD Reaction at Different Applied Electric Field Strengths^a^

**E** (V/Å)	pKw	Δ*F* (kJ/mol)	Δ*U* (kJ/mol)	Δ*S* (J/K mol)
0.00 (Exp.)	14.3 ± 0.1	82.5 ± 0.1	59.5 ± 0.1	–77.2 ± 0.5
0.00	14.7 ± 0.3	84.5 ± 1.5	53.0 ± 10.3	–96.1 ± 29.8
0.18	13.2 ± 0.3	75.8 ± 1.5	80.5 ± 10.3	13.5 ± 29.8
0.36	4.6 ± 0.3	26.6 ± 1.0	56.2 ± 6.3	89.7 ± 18.1
0.41	2.2 ± 0.2	13.7 ± 0.5		

a Experimental values are extracted from ref 54 pKw values are computed as, pKw = Δ*F*/(*RT* ln(10)) where *R* is the gas constant, and *T* is the temperature, 330 K.

In the absence of an applied field, the orientations of water molecules yield zero net polarization (see Figure 1c). Once the field is applied, the molecular dipoles progressively align with the field direction, with the degree of alignment increasing with field strength. This trend continues until dielectric saturation occurs at **E** = ∼0.25 V/Å, where the average reorientation reaches a plateau value. The decrease in pKw becomes more pronounced above approximately **E** = 0.2 V/Å, coinciding with the change in orientational behavior of water molecules shown in Figure 1c. The change in pKw under applied fields is governed by the free energy difference between products and reactants. Thus, it is not a direct consequence of how the system screens the electric field, but rather of the distinct ways in which reactants and products respond to it. However, a correlation between enhanced WD and molecular orientation indicates that water molecules in a highly ordered hydrogen-bond network become more susceptible to applied electric fields, pointing out a possible important entropic contribution to the thermodynamics of WD under strong fields.

### Field-Induced WD is Entropically Driven

To disentangle the entropic and energetic contributions of the WD reaction under electric fields, we performed simulations at multiple temperatures. Figure 2 shows the computed Helmholtz free energy between 300 and 390 K at three different field strengths. From these data, we extracted the reaction energy (Δ*U*) and reaction entropy (Δ*S*) by fitting to Δ*F* = Δ*U* – *T*Δ*S*. The results are summarized in Table 1. In the absence of electric fields, our simulations reproduce the experimental value, showing a large energetic cost (Δ*U* = 53.0 ± 10.3 kJ/mol) and substantial entropic penalty (Δ*S* = −96.1 ± 29.8 J/K mol).

**Figure 2:**
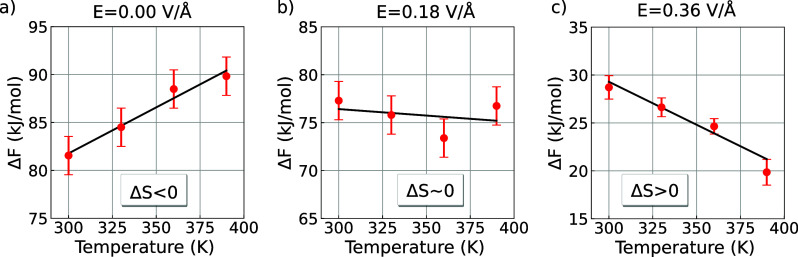
Dramatic change of temperature dependence of the WD reaction free energy under electric fields. Helmholtz free energy associated with the WD reaction as a function of temperature and selected values of electric field strengths.

At finite electric fields, while Δ*U* remains large and positive, there is a dramatic increase of Δ*S* by almost 200 J/K mol. To confirm these surprising results, we repeated the finite field simulation with another exchange–correlation functional, namely BLYP, and confirmed the presence of a large positive entropic contribution to the reaction free energy (see Figure S1). In the following sections, we examine the structural changes that help to rationalize this unexpected behavior.

### Weak and Moderate Electric Fields Strengthen the Hydrogen-Bond Network

We begin by analyzing the field strengths below **E** = 0.40 V/Å, where water dissociation remains a minor process. In Figure 3a-c, we present the O–O and O–H radial distribution functions. The application of an electric field shortens the O–O distance of neighboring waters (first peak of the gr_OO_), elongates the covalent intramolecular O–H bonds (first peak of the gr_OH_), and shortens the intermolecular H···O distances (second peak of the gr_OH_). These are all clear indications of the strengthening of the hydrogen-bond network with applied fields. This effect is further accompanied by the field-induced red shift of the O–H stretching vibrations, as shown in panel d.^55,56^ While not explicitly computed in this work, previous studies have shown that the molecular dipole moment of water increases linearly with the applied field intensity, resulting in an overall increase of approximately 10–15% in the average dipole moment magnitude when going from the zero-field regime to the predissociation field strength.^55^

**Figure 3:**
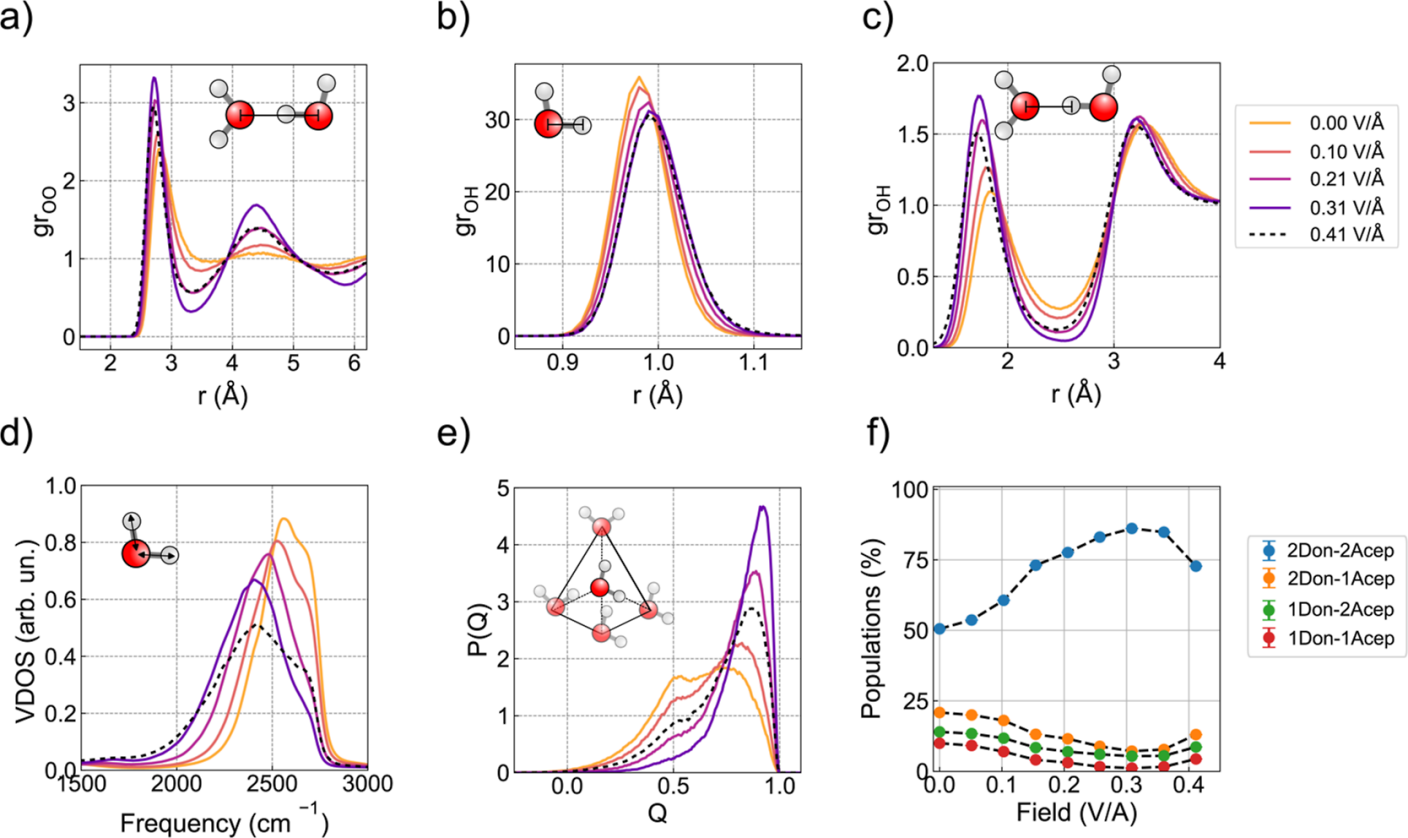
Water and hydrogen-bond network structure under electric fields. (a–c) Oxygen–oxygen and oxygen–hydrogen radial distribution functions, (d) vibrational density of states (VDOS) of water stretching mode, (e) tetrahedral order parameter, and (f) classification of water molecules according to the hydrogen bonding environment. Note that the main peak of the VDOS in panel d appears at approximately 2500 cm^–1^ due to the use of deuterium masses for hydrogen atoms.

To characterize the local angular order of the water molecules, we used the tetrahedral order parameter, *Q*.^57,58^ In its scaled form,^59^ *Q* varies from 0 for an ideal gas to 1 for a perfect tetrahedron. In Figure 3e, we present the corresponding *Q* distributions obtained at various fields. In the absence of an applied field, we observe a bimodal distribution consistent with previous simulations of water.^21,59^ As the field increases, the distribution shifts to the right, with the high-*Q* peak increasing at the expense of the low-*Q* shoulder, leading to a quasiisosbestic point at *Q* = 0.74. This trend reflects an increasing population of tetrahedrally coordinated water molecules, similar to structural changes observed upon cooling.^60,61^

Water molecules can be classified by the number of hydrogen bonds they donate (Don) and accept (Acep). For example, a water molecule that donates one hydrogen bond and accepts two is labeled as 1Don-2Acep. In Figure 3f, we show the evolution of the 2Don-2Acep, 2Don-1Acep, 1Don-2Acep, and 1Don-1Acep populations as a function of the field strength. Consistent with the behavior of *Q*, the 2Don-2Acep population increases from 0.50 at **E** = 0.00 V/Å to 0.84 at **E** = 0.36 V/Å. Thus, the electric field induces a continuous conversion of disordered configurations into more tetrahedral, ice-like structures.^62^ Such an “electrofreezing” effect has been proposed in theoretical studies,^63^ although its experimental verification remains elusive.^64^

The formation of new hydrogen bonds or the strengthening of existing ones leads to energy and mobility changes that directly translate into negative formation enthalpy and entropy. In our simulations, this value can be estimated from the temperature dependence of the equilibrium constant for the reaction, A + D ⇌ DA, where A and D, represent acceptor and donor, water molecules. Using the equilibrium constant computed at different temperatures, we obtain Δ*U* = −18.68 ± 2.05 kJ/mol and Δ*S* = −24.79 ± 5.83 J/K mol. These values are only marginally affected by small changes in the geometric criteria used to define a hydrogen bond (see Supporting Information for details). While hydrogen-bonding is just one of many factors contributing to the total entropy of an aqueous system, the high concentration of water in liquid water (55 mol/L) means that even small changes in the fraction of hydrogen-bonded molecules can have a significant thermodynamic impact. In this context, our results show that, prior to the onset of dissociation, the applied electric field drives the system toward a highly ordered, low-entropy state.

### Proton Defects Disrupt Hydrogen-Bond Network

At field strengths exceeding **E** = 0.36 V/Å, the concentration of ions surpasses 100 mM, significantly disrupting the hydrogen-bond network (dashed lines in Figure 3a–e), and therefore reversing the trends observed at lower field. Specifically, the height of the first peak of the gr_OO_ and the second peak of the gr_OH_ decreases, the *Q* distribution shows a lower tetrahedral order, and the number of 2Don-2Acep decreases. Together, these changes indicate that the formation of proton and hydroxide species weakens the overall hydrogen-bond network.

To confirm the disruptive effect of ions on water structuring at finite electric fields, we performed additional AIMD simulations on systems containing a permanent proton or hydroxide ion. Similar results were obtained for both types of systems, so for clarity, we discuss only the results for systems with a permanent proton. The field-induced water alignment with the applied field closely resembles that of pure water, indicating that the presence of ions has only a minor effect on the dielectric saturation (see Figure S8). However, when monitoring the average number of hydrogen bonds, a stark difference emerges. As shown in Figure 4a, in pure water, the number of hydrogen bonds steadily increases with field strength, consistent with the structural trends discussed earlier. In contrast, in the system containing an additional proton (blue curve in Figure 4a), the number of hydrogen bonds remains nearly constant across all field strengths. These results demonstrate that the presence of protons strongly inhibits the field-induced structuring of the hydrogen-bond network.

**Figure 4:**
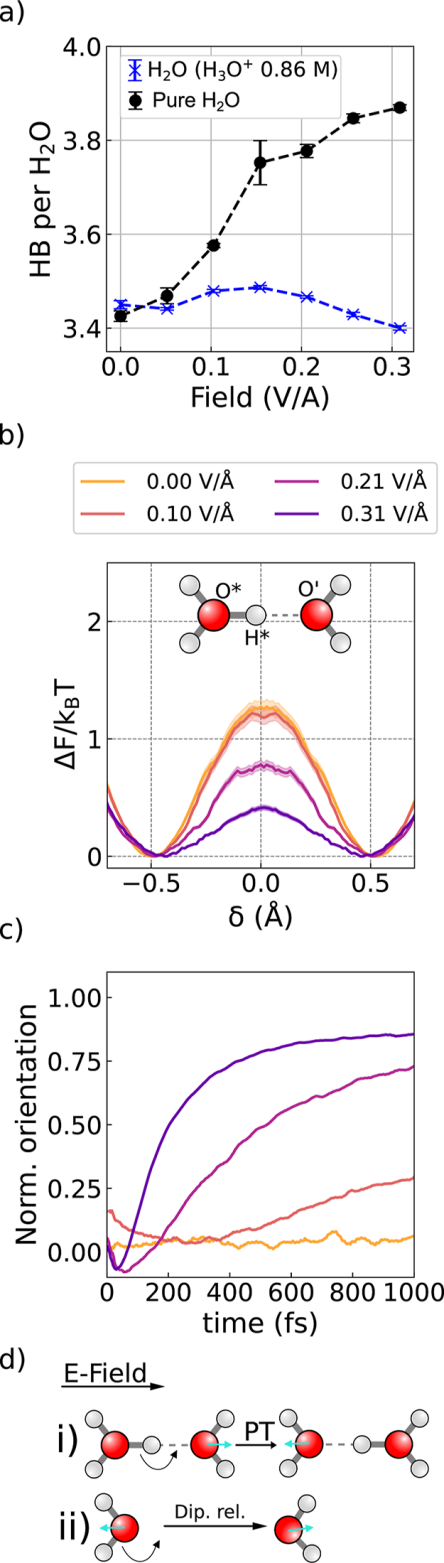
Proton conduction under electric fields disrupts the hydrogen-bond network. (a) Number of hydrogen bonds (HB) per water molecule for pure water and 0.86 M aqueous proton solution. (b) Proton transfer free energy barrier along the proton sharing coordinate, δ (see main text for its definition). (c) Average orientation of newly created water molecules after a proton transfer event. (d) Schematic representation of proton-induced water reorientation (i) Proton transfer (PT) event leads to a newly formed water molecule, which (ii) reorientates its dipole to align it parallel to the applied electric field (dipole relaxation). During this reorientation, existing hydrogen bonds are broken and new ones are formed.

To understand this disruptive effect, we calculated the proton transport free energy barrier. For this, we used the standard definition of the proton-sharing coordinate, δ = *d*_O*H*_ – *d*_O′H*_, where *d* denotes the distance between the respective atoms, and * and ′ refer to atoms belonging to the proton defect and nearest water molecule, respectively. For each frame, H* was selected to obtain the lowest of the possible δ. In the absence of an applied field, the computed proton transfer barrier is 1.2 *k*_B_ *T*, in reasonable agreement with previous AIMD simulations.^65,66^ At finite field strengths, the barrier decreases with increasing field, suggesting a faster proton diffusion.

Although faster proton diffusion explains the increased mobility, it is insufficient to account for the disruption of the hydrogen-bond network alone. Proton transfer events require a specific geometric arrangement of the hydronium ion, H_3_O^+^, and an acceptor water molecule. In Figure 4c, we present the average orientation of newly created water molecules, where we define time zero as the moment of the proton hop. Immediately after the proton transfer, the newly created water molecule exhibits no net orientation. Over the next few hundred femtoseconds, it reorients to align with the applied field, a process that induces the breaking of its preexisting hydrogen bonds. Thus, the field-enhanced proton transfer acts as a local, repeated disruption mechanism, progressively weakening the hydrogen-bond network and driving the system toward a more disordered, higher-entropy state (see schematic diagram in Figure 4d). This disruptive phenomenon was equally observed for simulations containing a permanent hydroxide ion (see Figure S9).

Putting together all these results, we are now in a position to rationalize the sign of the WD reaction entropy at large fields. This can be understood by comparing the thermodynamic states of the reactants (pure water) and the products (aqueous proton and hydroxide). At high fields, the reactant state becomes highly ordered: water molecules are strongly aligned with the field, the number of hydrogen bonds increases, and the hydrogen bonds are strengthened. Overall, the system is driven into a highly structured, low-entropy state. By contrast, the product state, due to the presence of disruptive ionic defects, exhibits a reduced orientational order, a lower number of hydrogen bonds, and weaker bonding. These changes contribute to an increase in rotational entropy. In addition, translational entropy rises, as one water molecule produces two highly mobile ionic species. The combined effect of these contributions gives rise to the large positive reaction entropy observed at high fields.

A precise quantification of the individual entropic components remains challenging and is still an active area of research.^67^ The formation entropy of a hydrogen bond has been estimated to be approximately −25 J/K mol,^68^ consistent with our own estimates presented in the Supporting Information. Based on this value, one would expect around four broken hydrogen bonds for the water dissociation reaction. However, an analysis of the data presented in Figure 4 suggests the disruption of approximately 15 hydrogen bonds. We attribute this apparent discrepancy to a possible reduction in the formation entropy of hydrogen bonds in the presence of electric fields. In a related study, Cassone et al. recently estimated rotational and translational entropy changes using classical and ab initio simulations via the two-phase thermodynamics (2PT) formalism.^62^ Their study found relatively small entropy reductions (a few J/K mol), although only field strengths below the dissociation threshold were considered. Their findings are consistent with our results and highlight the crucial role of ionic defects in altering the field-induced response of water.

The field-induced disruption mechanism proposed here resembles that suggested to explain the lower proton conductivity of ferroelectric ice XI compared to orientationally disordered ice Ih.^69^ In short, the reduced conductivity of ice XI arises from the difficulty in restoring dipole alignment due to the generation of proton traps:^70,71^ after each proton transfer event, the entire network of molecular dipoles must reorient to enable further transfers along a given path. At the same time, ice XI exhibits a lower onset field for WD than ice Ih. We hypothesize that such a greater propensity for the WD reaction stems from similar entropic effects to those described above.

Let us now turn to the reaction energy. Simple electrostatic arguments would predict that an external electric field stabilizes charge separation, thereby lowering Δ*U*. However, our results show that this is not the case. Instead, the molecular nature of the proton and hydroxide leads to a more complex scenario, in which compensating effects arise. Specifically, the same weakening and reduction of hydrogen-bonds that contribute to the positive Δ*S* also introduce a positive enthalpic contribution. This offsets the negative enthalpic contribution expected from charge stabilization, resulting in an overall relatively modest change in Δ*U* with increasing field strength. We believe that this competition may underlie the nonmonotonic dependence of Δ*U* reported in Table 1.

Proton defects are well-known to form strong hydrogen-bonds in water, leading to vibrational frequency shifts of several hundred cm^–1^, which arise in part from the strongly coupled vibrations of multiple water molecules solvating the defects.^72–74^ Recently, Car and co-workers demonstrated that structural correlations between proton defects and neighboring water molecules exhibit sharper peaks than correlations between water molecules themselves, indicating that the local environment around proton defects is, on average, more ordered.^51^ These findings are in agreement with the experimentally measured negative partial molar volumes for protons and hydroxide ions, confirming a more structured and compressed hydrogen-bond network near these ions.^75,76^ Moreover, the WD reaction itself is associated with a large negative reaction entropy, mainly originating from intermolecular interactions, further supporting the notion that proton defects act as structure makers under standard conditions. In contrast, our results show that under finite electric fields, proton and hydroxide ions behave as structure breakers. This demonstrates that an applied field can modulate and qualitatively transform ion–solvent interactions, turning classical structure makers into structure breakers. Future work will investigate whether this field-induced change in behavior also extends to other inorganic “spectator” ions.

## Conclusions

In this work, we presented AIMD simulations of bulk water under varying external electric fields and temperatures. Consistent with previous studies, we found that external fields act as structure-makers at low intensities and induce water autoionization at higher fields. We computed the reaction free energy, reaction internal energy (Δ*U*), and reaction entropy (Δ*S*) associated with the WD reaction, showing that the reaction is entropically hindered in the absence of a field but becomes entropy-driven under finite fields. Furthermore, through additional simulations involving proton and hydroxide ions, we demonstrated that proton defects inhibit the field-induced water structuring by randomizing molecular orientations during proton transfer events, thereby weakening the hydrogen-bond network.

Our AIMD simulations are based on DFT employing the revPBE-D3 exchange correlation functional. This functional belongs to the generalized gradient approximation (GGA) family and is known to overestimate hydrogen-bond strengths and underestimate hydrogen transfer barriers.^21,65,77,78^ Additionally, while the inclusion of nuclear quantum effects (NQEs) further modulates the strength of the hydrogen-bond network and lowers the field-induced dissociation threshold,^31^ it is well-known that combining NQEs with a GGA-level potential energy surface leads to a drastic overestimation of these effects.^65,77,79^ Thus, omitting NQEs at the GGA level often results in better accuracy due to a partial cancellation of errors. Indeed, this likely explains why our simulations yield a surprisingly accurate value of pKw (see Table 1). To go beyond these limitations, the use of machine learning interatomic potentials (MLIPs) is required. Recent developments in that area have enabled large-scale simulations using hybrid and meta-GGA functionals,^21,51^ as well as explicitly correlated methods,^80–82^ and new approaches that allow coupling to external fields have been proposed.^56,83–85^ However, in most cases, the architecture is based on zero-field expansion or relies on a reference frame, limiting their ability to capture water dissociation under strong fields. Our group is actively developing next-generation machine learning interatomic potentials (MLIPs) designed to enable accurate simulations of field-driven dissociation processes. These models will allow us to gain in-depth mechanistic insights, investigate time-dependent properties, and study more complex systems with higher accuracy and over extended time scales in the future.

Despite the simplicity of our system, the decisive role of field-induced solvent ordering observed for the WD reaction could have important implications in other contexts, such as the hydrogen evolution reaction (HER), where rates depend sensitively on the ordering of interfacial water molecules under electric fields.^86,87^ In most cases, such rate enhancements are rationalized in terms of purely energetic stabilization of transition-state geometries. However, recent studies suggest that increased activation entropy may play a crucial role in voltage-driven water dissociation (WD) catalysis. For example, Chen et al. investigated the temperature dependence of the WD reaction in bipolar membrane junctions with TiO_2_ catalysts.^88^ By fitting their results to an Arrhenius-like expression, they found that the activation energy remained independent of the applied overpotential, while the preexponential factor increased with increasing overpotential. Based on transition state theory, the authors attributed this change in the prefactor to an increase in activation entropy. In a related study, similar behavior was observed across a range of oxide catalysts, and was further linked to the bias-dependent dispersion of interfacial capacitance.^89^ More recently, large entropic effects associated with the structuring of interfacial water or solvent molecules have been reported to govern reaction rates in a variety of electrochemical processes, including the hydrogen evolution reaction (HER) on platinum-group and coinage metals in acidic media,^90^ CO and CO_2_ electroreduction,^91,92^ and ammonia oxidation.^93^ Our simulations provide strong evidence for the emerging view that field-induced molecular organization and solvent alignment, namely entropic effects, may be central to accelerate numerous reactions at aqueous interfaces,^14,92,94,95^ highlighting the need for more temperature-dependent studies of interfacial aqueous reactions. Clearly, a more rigorous understanding will require approaches that capture macroscopic length and time scales. We hope that our results can inform ongoing mean-field and continuum-scale modeling efforts aimed at describing these complex scenarios more accurately.^96,97^

In conclusion, our work reveals the underlying thermodynamic factors that govern arguably the simplest field-induced aqueous reaction, the WD reaction. Given the parallels with processes relevant to atmospheric chemistry, electrochemistry, and biochemistry, we propose that entropic effects are likely more ubiquitous and influential than previously recognized. These insights could prove crucial in the rational design of novel aqueous catalysts for “on-water” and electrochemical reactions.

## Methods

The pure water system consisted of 64 H_2_O molecules in a cubic box with periodic boundary conditions and a side length of 12.42 Å. Aqueous proton (hydroxide) systems were generated by replacing a water molecule with a single H_3_O^+^ (or OH^–^) in the same setup, resulting in 63 H_2_O + 1H_3_O^+^ (or 63 H_2_O + 1 OH^–^). In the Supporting Information, we provide the results of additional simulations with 128 H_2_O molecules (and 127 H_2_O + 1H_3_O^+^/1 OH^–^) in a box with a side length of 15.644 Å to assess finite-size effects. Consistent with previous studies using comparable methodology,^22,31^ the sensitivity of the computed reaction free energy to the system size was found to be relatively small, within a few kcal/mol.

AIMD simulations were performed with the CP2K code,^98^ using the revPBE exchange correlation functional^45^ augmented with D3 dispersion corrections.^46^ Kohn–Sham orbitals were expanded in a TZV2P basis set, and the electron density was represented with an auxiliary plane-wave basis with a cutoff of 400 Ry. This setup has been shown to reproduce the structural and dynamical properties of water reasonably well.^99^ Hydrogen atoms were assigned a mass of 2.0 amu, and a time step of 2.0 fs was used to integrate the equations of motion. Simulations were carried out in the NVT ensemble using the stochastic velocity rescaling thermostat^100^ with a 50 fs time constant, discarding the first 10 ps for thermalization. Unless otherwise stated, trajectories were run for 100 ps after equilibration (see convergence tests in Figures S10 and S11). Error bars were computed by dividing the trajectory into ten 10 ps blocks and calculating the standard deviation of the mean. To obtain Δ*S* and Δ*U*, simulations were run at 300, 330, 360, and 390 K. We note that the highest temperature corresponds to a superheated regime of water. Simulations under external electric fields were initialized from prethermalized structures at lower field strengths. To ensure numerical stability, the field was increased incrementally in steps of 0.001 hartree/Bohr (∼0.05 V/Å).

Umbrella sampling simulations for the calculation of pKw closely followed the methodology reported by Joutsuka.^22^ These simulations were performed using i-PI^101^ coupled to CP2K^98^ and PLUMED.^102^

## Supplementary Material



## Data Availability

All data required to reproduce the findings of this work is available at https://github.com/water-ice-group/WD_field_I.

## Notes

Details on the enhanced sampling simulations, system size and simulation time convergence tests, comparisons with other exchange–correlation functionals, temperature dependence of hydrogen-bond formation, and further analysis of systems containing OH^–^ (PDF)
