# Supplementary material for: Entropy Governs the Structure and Reactivity of Water Dissociation Under Electric Fields
Source: J Am Chem Soc. 2025 Sep 25;147(49):44885–94. doi: 10.1021/jacs.5c12397 (PMC12703742; doi:10.1021/jacs.5c12397)
Supplement: Supplementary file 1 [file ja5c12397_si_001.pdf]

Supporting Information for: Entropy governs the structure and reactivity of water dissociation under electric fields

Yair Litman*,†,‡ and Angelos Michaelides*,†

†Yusuf Hamied Department of Chemistry, University of Cambridge, Lensfield Road, Cambridge, CB2 1EW, UK

‡Max Planck Institute for Polymer Research, Ackermannweg 10, 55128 Mainz, Germany

E-mail: litmany@mpip-mainz.mpg.de; am452@cam.ac.uk

Enhanced Sampling Simulations

Free energy calculations of the WD reaction at $\mathbf{E} = 0.00$ V/Å and $\mathbf{E} = 0.18$ V/Å required enhanced sampling techniques. We employed umbrella integration,[1] which combines thermodynamic integration and umbrella sampling to reduce statistical errors. Simulations were performed using i-PI[2] coupled to CP2K[3] and PLUMED.[4] As a collective variable, we used the coordination number of a selected oxygen atom, defined as:

$$n_{\text{cov}} = \sum_{i \in H} \frac{1 - \left(\frac{r_i}{R_0}\right)^{12}}{1 - \left(\frac{r_i}{R_0}\right)^{24}}, \tag{1}$$

where the sum runs over all hydrogen atoms, $R_0 = 1.38$ Å, and r_i is the distance between the selected oxygen atom and the i-th hydrogen. Umbrella integration used 11 windows spanning n_{cov} values from 2.0 to 1.0, with a harmonic bias of 200 kcal/mol.[5,6] We used

$n_{\mathrm{cov}} = 2.0$ and $n_{\mathrm{cov}} = 1.2$, to define the intact water and hydroxide states, respectively.[7] Although the need for a second collective variable[5,6] or more sophisticated ones based on Voronoi tesselation[8,9] has been suggested, the limited simulation times in AIMD mean that these refinements would be inconsequential relative to our statistical error bars.

Additional convergence and robustness tests

To assess the sensitivity of our results to the specific choice of exchange–correlation functional, we repeated the calculation using a different GGA functional. In Fig. 1, we present the free energy of the water dissociation reaction at different temperatures at $\mathbf{E} = 0.36$ V/Å obtained with the BLYP-D3 functional. The fitted values of ΔU and ΔS are 20–25% smaller in magnitude compared to those obtained with revPBE-D3 presented in the main text. Importantly, in both cases, ΔS remains large and positive, demonstrating that our conclusions are robust within this level of theory. Because classical nuclei simulations using GGA functionals are known to be benefited by the partial error cancelation arising from the neglect of nuclear quantum effects and the underestimation of the hydrogen transfer barrier,[10,11] we do not anticipate qualitative changes if more accurate simulations are performed, for example employing hybrid or meta-GGA functionals and including nuclear quantum effects.

Fig. 2 shows the time traces of the umbrella simulations for a selected system. It can be observed that the reaction coordinate is adequately sampled between the reactant and product states.

To verify the small impact of finite size effects reported in Ref.,[12] we repeated some simulations, doubling the volume of the simulation box. We used a 15.644 $Å^3$ simulation box containing 127 H_2O molecules and 1 H_3O^+ or 1 OH^-. Fig. 3 and 4 show the proton transfer free energy profiles for systems containing a permanent proton and hydroxide ion, respectively. In both cases, simulations with 64 and 128 oxygen atoms yield comparable energy profiles. Fig. 5, shows the average number of hydrogen bonds per water molecule at

different external field strengths for pure water and aqueous proton in the small and large box sizes. In both scenarios, the proton exhibits a pronounced and comparable structure-breaking effect.

Fig. 6 and 7 show the temperature dependence of the free energy associated with hydrogen bond formation, using slightly different angle and distance criteria, respectively. Tab. 1 presents the corresponding formation energies and entropies, showing that variations in the hydrogen bond definition have only a minimal impact on its thermodynamics.

Fig. 8 shows the field-induced water alignment for pure water and acid and basic solutions, where it is direct to observe that the presence of ions has only a minor effect on the dielectric saturation.

Fig. 9 presents the number of hydrogen bonds, the proton transfer free energy barrier, and the relaxation dynamics of newly formed water molecules for a system containing an OH^-. Similar to that reported for H_3O^+ in the main text, OH^- exhibits a markedly structure-breaking behavior. However, when comparing with the results for proton solutions shown in Fig. 4a of the main text, it is evident that proton solutions exhibit a slightly lower number of hydrogen bonds per water molecule than the corresponding hydroxide solutions. We attribute this subtle difference to the distinct molecular geometries of the ions. More specifically, hydroxide ions, with their linear structure, align more easily with the electric field, while the three-dimensional geometry of the hydronium ion is less compatible with the oriented hydrogen-bond network.

Fig. 10 shows the cumulative average of ions for two representative simulations at finite fields, demonstrating that the simulation times used are sufficient to support the claims presented in the main text. Fig. 11 shows the corresponding figure for simulations in the absence of external fields.

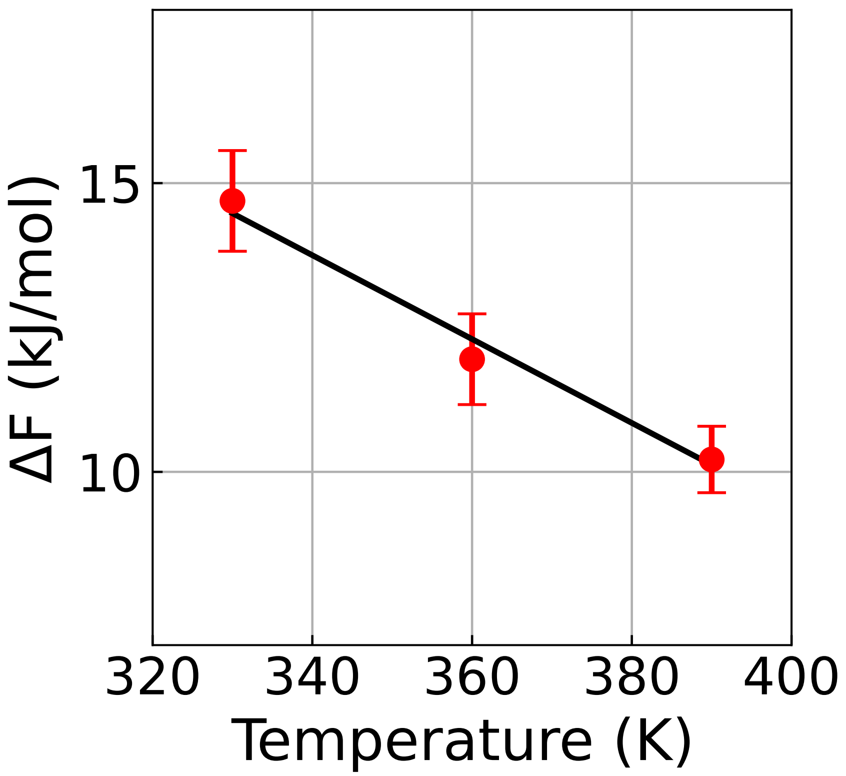

Figure 1: Helmholtz free energy of the water dissociation reaction as a function of temperature obtained from AIMD simulations with BLYP-D3 exchange correlation functional at $\mathbf{E}$=0.36 V/Å. Linear fit of the data leads to ΔU= 38.44 ± 6.28 kJ/mol and ΔS=72.61 ± 16.99 J/mol K.

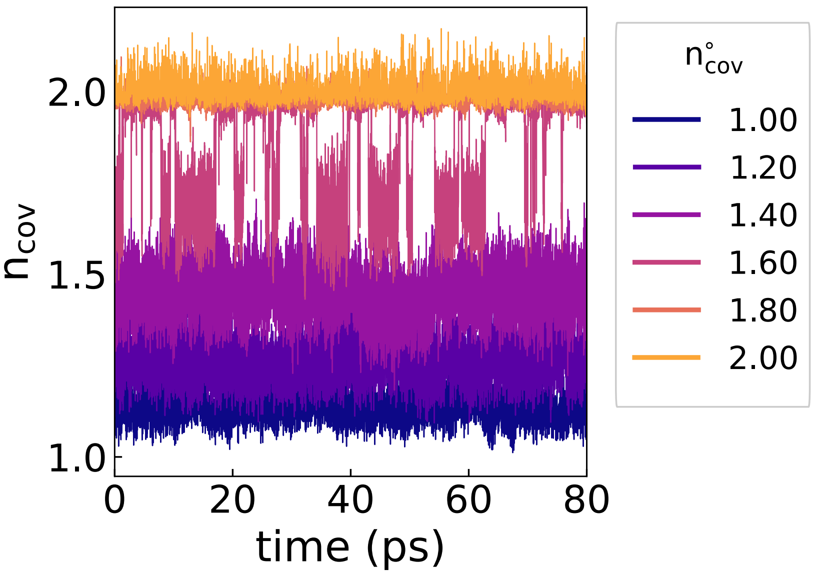

Figure 2: Time traces of selected umbrella windows obtained from revPBE-D3 simulations at 330 K in the absence of an external field. n°_{cov} corresponds to the coordination number value set for each umbrella window, and n_{cov} represents the instantaneous coordination number. For clarity, only every second umbrella window is shown.

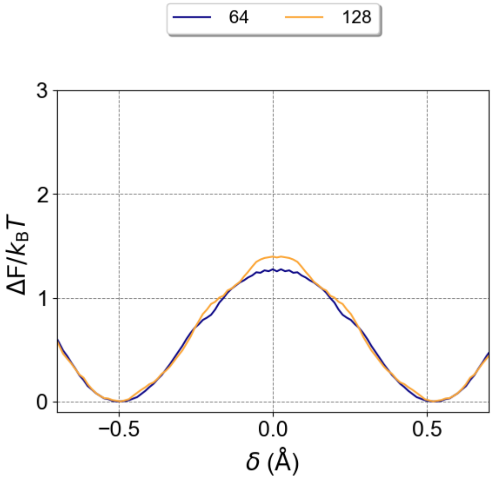

Figure 3: Proton transfer free energy along the proton sharing coordinate for a system containing 64 oxygen atoms (63 H_2O + 1 H_3O^+) and 128 oxygen atoms (127 H_2O + 1 H_3O^+)

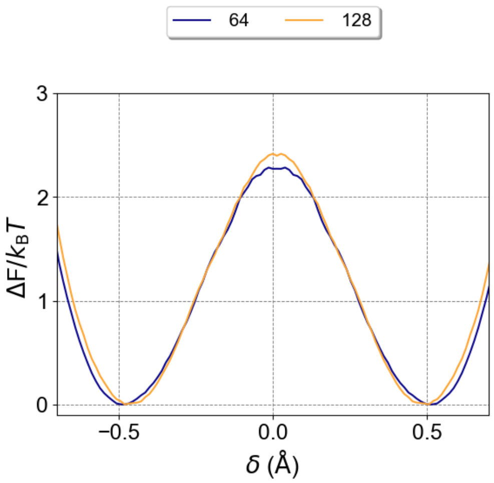

Figure 4: Proton transfer free energy along the proton sharing coordinate for a system containing 64 oxygen atoms (63 H_2O + 1 OH^-) and 128 oxygen atoms (127 H_2O + 1 OH^-).

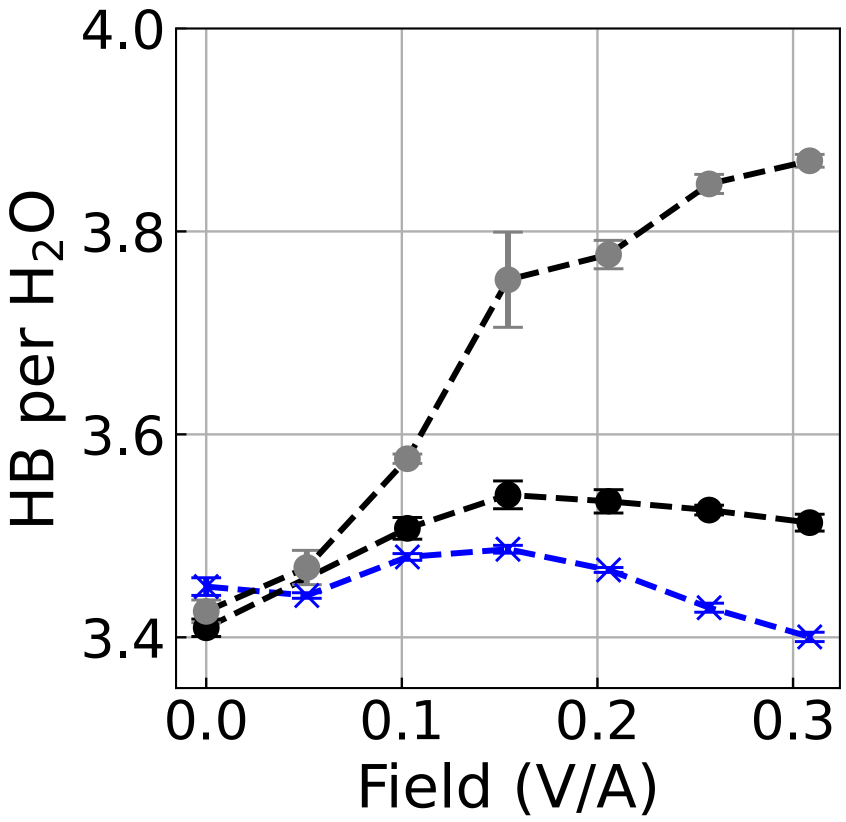

Figure 5: Number of hydrogen-bonds (HB) per water molecule for pure water (gray circles) and aqueous proton solution for boxes containing 64 oxygen atoms, 63 H_2O + 1 H_3O^+, and 128 oxygen atoms, 127 H_2O + 1 H_3O^+ (blue crosses and black circles respectively)

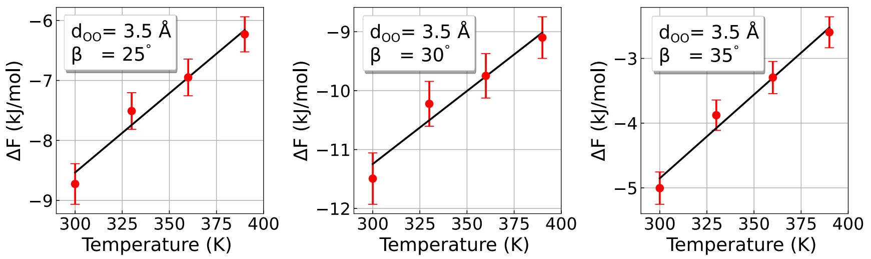

Figure 6: Temperature dependence of the hydrogen-bond formation free energy for different angle definitions

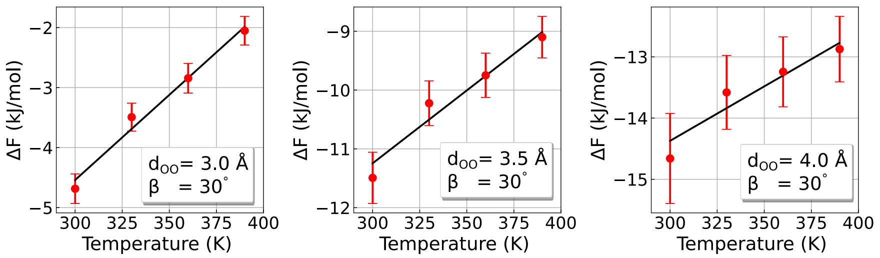

Figure 7: Temperature dependence of the hydrogen-bond formation free energy for different distance definitions. Fitted values for ΔU and ΔS are reported in Tab. 1. Fitted values for ΔU and ΔS are reported in Tab. 1.

Table 1: Formation energy and entropy of hydrogen bond using different distance, d_{OO}, and angle, β, thresholds.

d_{OO}	β	ΔU (kJ/mol)	ΔS (J/ K mol)
30.00	3.00	-13.05 ± 1.25	-28.38 ± 3.61
30.00	3.50	-18.68 ± 2.05	-24.79 ± 5.83
30.00	4.00	-19.71 ± 3.28	-17.78 ± 9.29
25.00	3.50	-16.45 ± 1.64	-26.38 ± 4.68
30.00	3.50	-18.68 ± 2.05	-24.79 ± 5.83
35.00	3.50	-21.80 ± 3.11	-23.92 ± 8.77

References

(1) Kästner, J.; Thiel, W. Bridging the gap between thermodynamic integration and umbrella sampling provides a novel analysis method: "Umbrella integration". *The Journal of Chemical Physics* **2005**, *123*, 144104.

(2) Litman, Y. et al. i-PI 3.0: A flexible and efficient framework for advanced atomistic simulations. *The Journal of Chemical Physics* **2024**, *161*, 062504.

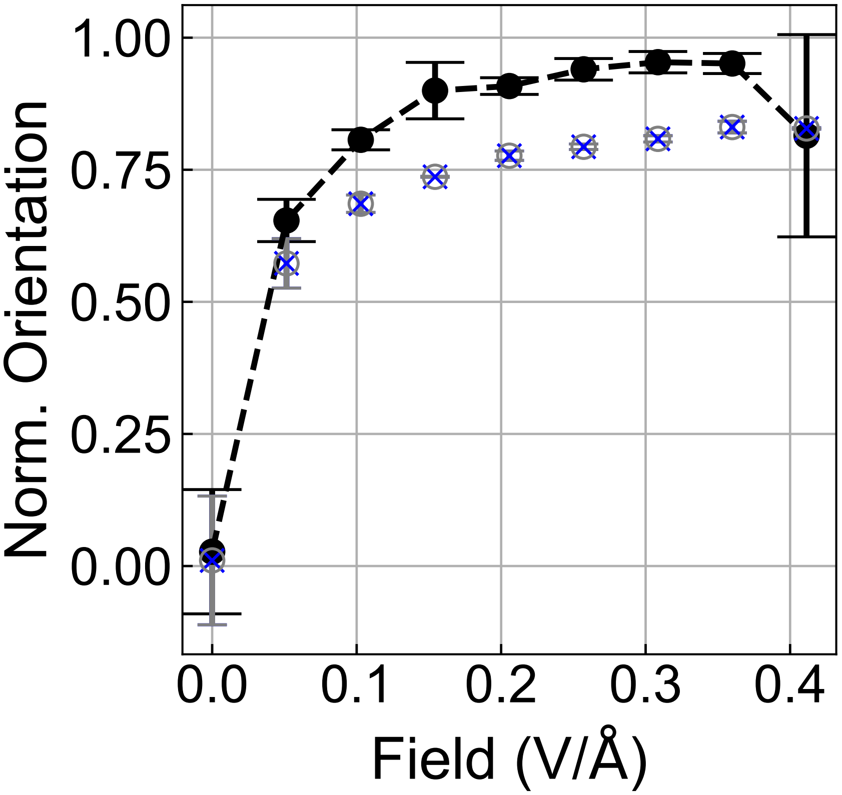

Figure 8: Average orientation of molecular dipoles along the field direction for pure water (black filled circles), aqueous proton (blue crosses), and aqueous hydroxide (gray empty circles). A value of 1.00 represents a perfect alignment between the molecular dipoles and the external field.

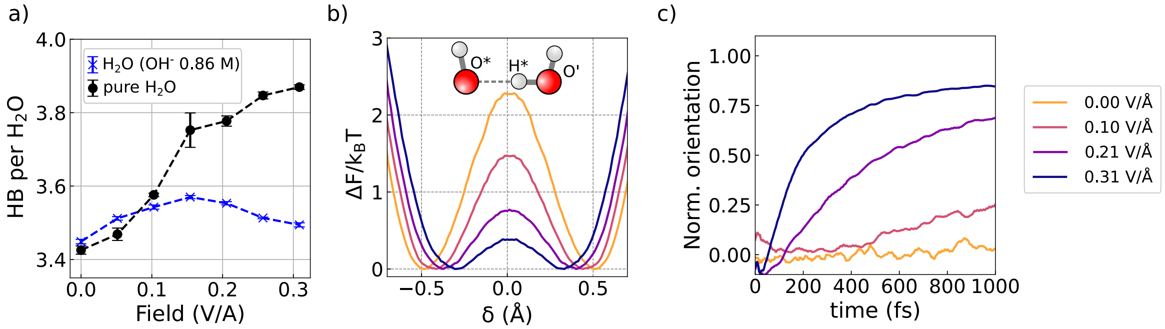

Figure 9: a) Number of hydrogen-bonds (HB) per water molecule for pure water and 0.86 M aqueous hydroxide solution. b) Proton transfer free energy barrier along the proton sharing coordinate, δ (see main text for its definition). c) Dipole relaxation of the newly formed water molecule after a proton transfer event.

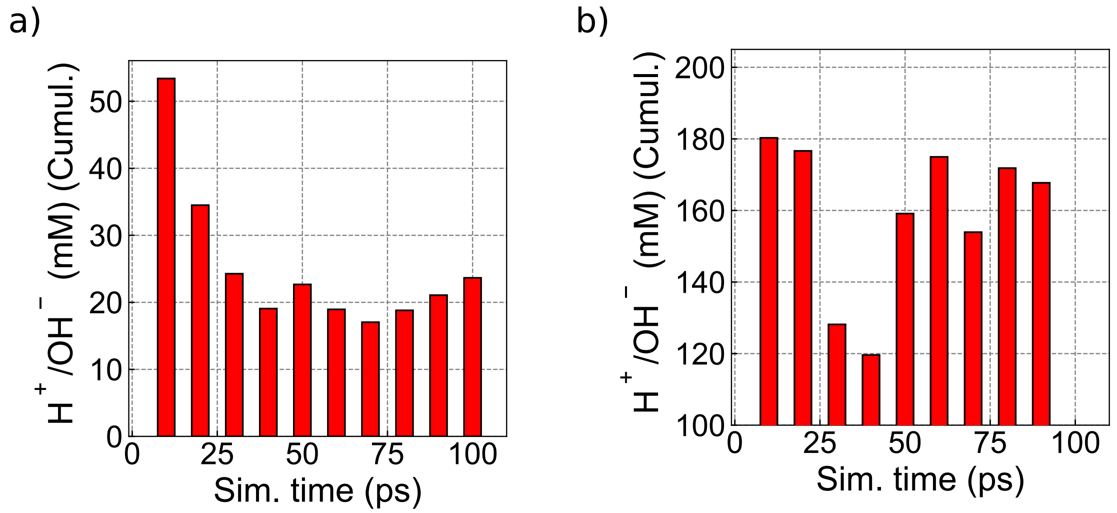

Figure 10: a) Cumulative average of the proton defect concentration at 330 K with a) $\mathbf{E} = 0.36$ V/Å and b) $\mathbf{E} = 0.41$ V/Å.

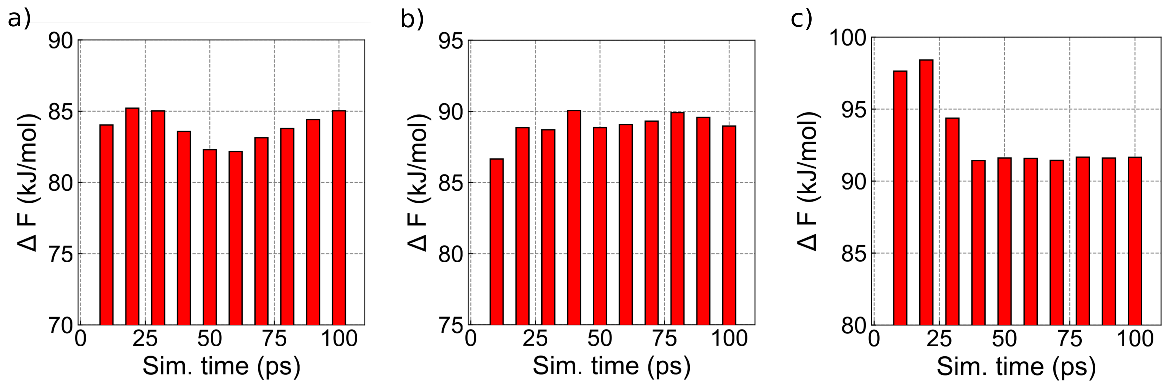

Figure 11: Cumulative average of reaction free energy in the absence of field at a) 330 K, b) 360 K, and c) 390 K.

(3) Kühne, T. D. et al. CP2K: An electronic structure and molecular dynamics software package - Quickstep: Efficient and accurate electronic structure calculations. *The Journal of Chemical Physics* **2020**, *152*, 194103.

(4) Bonomi, M. et al. Promoting transparency and reproducibility in enhanced molecular simulations. *Nature Methods* **2019**, *16*, 670–673.

(5) Joutsuka, T. Molecular Mechanism of Autodissociation in Liquid Water: Ab Initio Molecular Dynamics Simulations. *The Journal of Physical Chemistry B* **2022**, *126*, 4565–4571.

(6) Dasgupta, S.; Cassone, G.; Paesani, F. Nuclear Quantum Effects and the Grotthuss Mechanism Dictate the pH of Liquid Water. *The Journal of Physical Chemistry Letters* **2025**, *16*, 2996–3003.

(7) de la Puente, M.; Laage, D. How the Acidity of Water Droplets and Films Is Controlled by the Air–Water Interface. *Journal of the American Chemical Society* **2023**, *145*, 25186–25194.

(8) Grifoni, E.; Piccini, G.; Parrinello, M. Microscopic description of acid–base equilibrium. *Proceedings of the National Academy of Sciences* **2019**, *116*, 4054–4057.

(9) Andrade, M. C.; Car, R.; Selloni, A. Probing the self-ionization of liquid water with ab initio deep potential molecular dynamics. *Proceedings of the National Academy of Sciences* **2023**, *120*, e2302468120.

(10) Ceriotti, M.; Fang, W.; Kusalik, P. G.; McKenzie, R. H.; Michaelides, A.; Morales, M. A.; Markland, T. E. Nuclear Quantum Effects in Water and Aqueous Systems: Experiment, Theory, and Current Challenges. *Chemical Reviews* **2016**, *116*, 7529–7550.

(11) Atsango, A. O.; Morawietz, T.; Marsalek, O.; Markland, T. E. Developing machine-learned potentials to simultaneously capture the dynamics of excess protons and hydroxide ions in classical and path integral simulations. *The Journal of Chemical Physics* **2023**, *159*, 074101.

(12) Cassone, G. Nuclear Quantum Effects Largely Influence Molecular Dissociation and Proton Transfer in Liquid Water under an Electric Field. *The Journal of Physical Chemistry Letters* **2020**, *11*, 8983–8988.
